# Amphiphilic drug interactions with model cellular membranes are influenced by lipid chain-melting temperature

**DOI:** 10.1098/rsif.2013.1062

**Published:** 2014-05-06

**Authors:** Duncan Casey, Kalypso Charalambous, Antony Gee, Robert V. Law, Oscar Ces

**Affiliations:** 1 Institute of Chemical Biology, Imperial College, Exhibition Road, London, UK; 2 School of Biochemistry, University of Bristol, Bristol, UK; 3 Imaging Sciences and Biomedical Engineering, King's College, London, UK

**Keywords:** membrane biophysics, drug transport, lipid hydrolysis, high-performance liquid chromatography

## Abstract

Small-molecule amphiphilic species such as many drug molecules frequently exhibit low-to-negligible aqueous solubility, and generally have no identified transport proteins assisting their distribution, yet are able to rapidly penetrate significant distances into patient tissue and even cross the blood–brain barrier. Previous work has identified a mechanism of translocation driven by acid-catalysed lipid hydrolysis of biological membranes, a process which is catalysed by the presence of cationic amphiphilic drug molecules. In this study, the interactions of raclopride, a model amphiphilic drug, were investigated with mixtures of biologically relevant lipids across a range of compositions, revealing the influence of the chain-melting temperature of the lipids upon the rate of acyl hydrolysis.

## 1. Introduction

The interplay between the majority of small-molecule compounds and biological membranes remains poorly understood, despite intense investment and research from both academia and industry. Many drugs display poor aqueous solubility, leading to their absorption by lipid bodies such as cell membranes, adipose tissue and liposomes *in vivo*, which may alter the transport kinetics of the compound, the stability and integrity of the lipid assembly, or both. These relationships are of critical importance when attempting to understand, predict or modulate the pharmacokinetics and availability of these compounds.

Hydrophobic and amphiphilic substances such as drug molecules can traverse the bilayer either by simple diffusion or by hijacking the cell's transport system [1]. Membrane proteins (including species such as carriers and channels) mediate substrate flux by facilitated or active transport in the direction of or against concentration gradients, the latter of which is energy dependent. Energy is used either in the form of ATP hydrolysis [2], known as primary active transport, or through the dissipation of electrochemical gradients by coupling transport to secondary molecules, classified as secondary active transport [3]. In recent years, these proteins have been the focus of clinical attention owing to their association with multidrug resistance [4].

Unfavourable drug interactions with proteins can have adverse effects, including drug resistance and major side effects through low drug specificity. However, drug–protein interactions are not the only consideration during drug design: the extent of membrane partitioning of a drug is a key physico-chemical property that must be considered. The partition coefficient (log *P*) and distribution function (log *D*) of compounds are often used as crude measures of the distribution of compounds *in vivo* [5], as Overton's rule suggests that lipophilic compounds will traverse a lipid bilayer faster than more hydrophilic species [6]. Unfortunately, while the behaviour of some drug compounds correlates well with log *P* and log *D* values, there are also many exceptions [7], as these parameters in combination with specific active processes can lead to the sequestration of drugs within membranes and other non-target tissue. This results in reduced efficacy in therapeutic uses, leading to increased doses and subsequent toxicity as well as severely limiting contrast in applications such as medical imaging.

**Figure 1.**
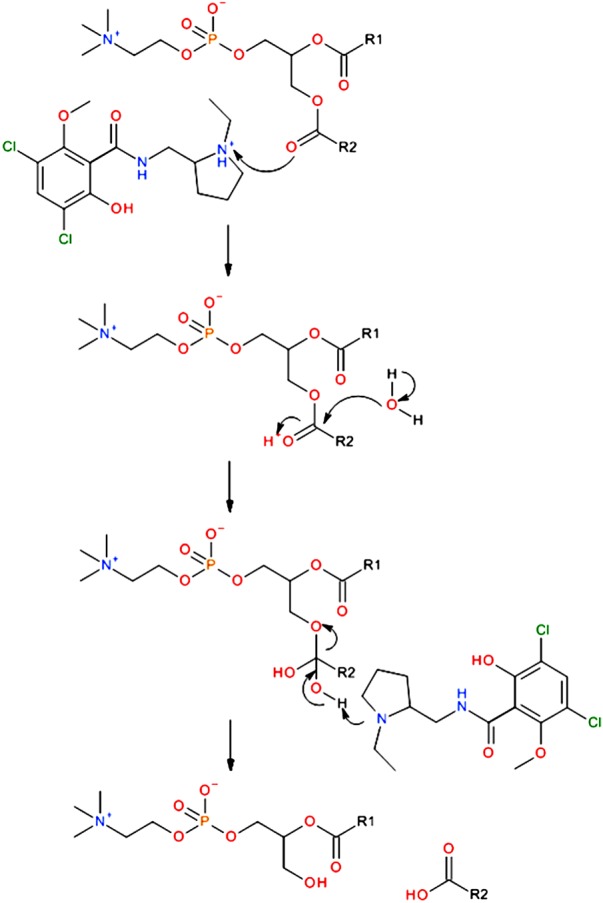
The mechanism of lipid hydrolysis catalysed by the presence of an amphiphilic species acting as a phase transfer agent, illustrated here with a phosphatidylcholine species and the CAD raclopride, where R1 and R2 are alkyl chains. As the reaction progresses, the diacyl species which promote the formation of lipid bilayers are replaced by highly curved lysolipids and fatty acids which prefer to form curved interfaces, leading to dramatic perturbations within the membrane. (Online version in colour.)

It is clear that this sequestration of material in non-target tissues, often referred to as non-specific binding, is a far more complex process than has been previously thought and that protein-mediated drug transport alone cannot adequately describe the translocation of bulky hydrophobic or amphiphilic molecules not recognized by the cell (e.g. the cationic amphiphilic drugs; CADs) [8]. In the light of this, other modes of drug uptake based on direct chemical or physical interactions between the drug and lipid molecules have been postulated. Baciu *et al*. [9] demonstrated the CAD-catalysed hydrolysis of a phosphatidylcholine membrane via its lipid ester bonds, generating a molecule of mono-acyl phosphatidylcholine and the concomitant fatty acid as shown in figure 1. This process rapidly causes dramatic perturbations to the membrane topography, as degradation products begin to accumulate. Fluorescence microscopy studies have revealed that drug-bearing membrane fragments are ejected from the membrane within seconds of the compound's administration.

Here, we present studies describing lipid hydrolysis by the dopamine-D2 antagonist raclopride tartrate as a function of membrane composition. Previous studies have focused upon model systems comprising pure lipid species predominantly in 1,2-dioleoyl-*sn*-glycero-3-phosphatidylcholine (DOPC), as it adopts the fluid lamellar phase over a wide range of hydrations and temperatures. Model membranes such as these, comprising a small number of synthetic and purified lipids, are routinely used to mimic the behaviour of biological membranes *in vitro* [10]. These systems are generally protein free and provide a relatively stable platform with physical characteristics similar to those found in most cells, allowing the direct and quantitative analysis of phenomena in a membrane environment. Although these models are simplistic compared with biological membranes, they are currently the only practical option for *in vitro* characterization of membrane properties.

**Figure 2.**
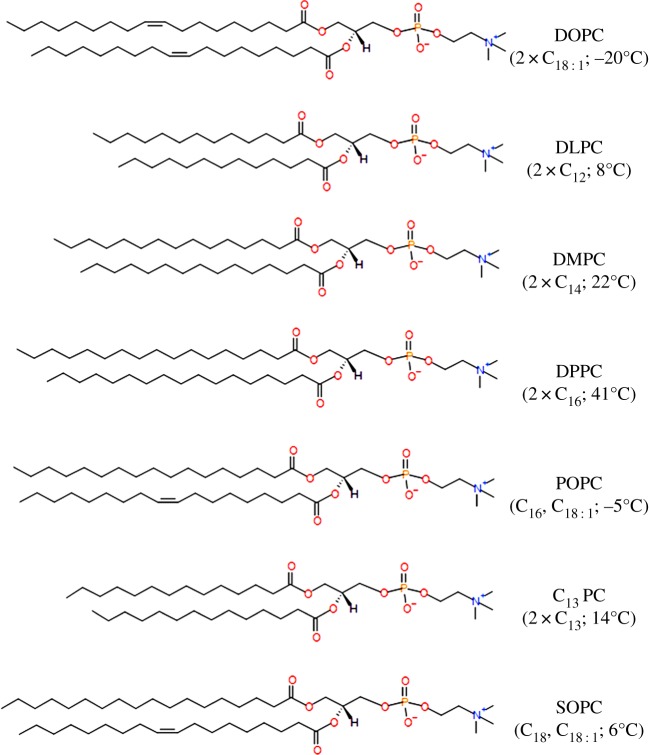
The structures of 1,2-dioleoyl-*sn*-glycero-3-phosphatidylcholine (DOPC), 1,2-dilauroyl-*sn*-glycero-3-phosphocholine (DLPC), 1,2-dimyristoyl-*sn*-glycero-3-phosphocholine (DMPC), 1,2-palmitoyl-*sn*-glycerol-3-phosphocholine (DPPC), 1-palmitoyl-2-oleoyl-*sn*-glycero-3-phosphocholine (POPC), 1,2-tridecanoyl-*sn*-glycero-3-phosphatidylcholine (C_13_ PC) and 1-stearoyl-2-oleoyl-*sn*-glycero-3-phosphatidylcholine (SOPC). Carbon chain lengths and melting transition temperatures given in parentheses. (Online version in colour.)

To isolate and characterize the influence of the chain packing within a membrane upon lipid hydrolysis mediated by raclopride tartrate, saturated and mix-tailed phosphatidylcholine lipids were systematically doped into DOPC bilayers, and the rate(s) of hydrolysis was measured. The structures of all lipids used are shown in figure 2.

For the first time, to the best of our knowledge, we can demonstrate direct dependence of the rate of reaction upon the melting temperature of the lipid, and thus the efficiency of fatty chain packing interactions within the system.

## 2. Material and methods

Lipids were purchased from Avanti Lipids (AL, USA) at the highest purity available. All other compounds were purchased from Sigma–Aldrich (UK), unless otherwise specified and were of at least 95% purity.

### 2.1. Lipid preparation

Sample preparation was adapted from previously reported studies [11]. In each vial, 25 μmol (≈20 mg) of lipid or lipid mixtures with 5 mol% drug was dissolved in a mixture of 1 : 1 chloroform and methanol, and the solvent removed under a nitrogen stream. The lipids or lipid mixtures were then dried under vacuum overnight, before being hydrated with 60 μl 10 mM phosphate-buffered saline (PBS) at pH 7.4 to a final concentration of 0.42 M. The assay vials were then vortexed, and centrifuged three times to ensure homogeneity and incubated at 37°C throughout the course of the experiments in aluminium heating blocks to prevent the formation of a thermal gradient in the vials. All buffers were made using high-performance liquid chromatography (HPLC)-grade water (VWR, UK). Samples (6 μl) were taken at 3–4 day intervals, and dispensed into a 1 ml HPLC sample vial. These samples were dried under vacuum in a desiccator to halt any further reaction, and were then redissolved into 1 ml of methanol for HPLC analysis.

### 2.2. HPLC conditions

Analyses were performed using an all-polyetheretherketone (PEEK), dual-pump system (model 626 LC, Waters). Measurements were conducted on an experimental 150 × 3.9 mm Agilent PLRP-AQ column (packed with 12 μm hydrophilic, hydroxylated polymer beads with hydrophobic pores of around 100 Å and a pore volume of 45%) and analysed via evaporative light scattering using an ESA 301 detector. The solvent profile consisted of a short run at 95% water to clear the buffer and inorganic salts from the column (which were otherwise found to co-elute with the lysolipids), followed by a rapid climb to 70% methanol and then a shallow gradient to 95% methanol. Eluting solvents were degassed through a Degassex DG-401 unit (Phenomenex). Methanol (25 μl) solution was injected on each occasion using a Waters 771 Plus autosampler module, giving strong evaporative light-scattering detector (ELSD) signals with a good signal-to-noise ratio. Calibration was conducted using standard solutions of each lipid in methanol to give injections of 0.001–0.1 mM. The mass injected was plotted against the peak area recorded by the ELSD. There was no significant change in ELSD signal between identical concentrations of the different species injected, allowing the hydrolysis data to be collected as a simple percentage.

### 2.3. Small-angle X-ray scattering

Small-angle X-ray scattering (SAXS) was conducted using custom-built equipment, based around a Bede (Durham, UK) Microsource X-ray generator with integrated optics, an Ealing (Ealing Electro Optics, UK) linear transition stage and a Photonic Science Ltd (Battle, UK) Gemstar intensified CCD X-ray detector [12]. X-ray data were acquired over a 60 s exposure, and spacings were calibrated against a silver behenate standard [13]. Sample diffraction patterns were analysed with the IDL-based AXcess software package that was developed by Andrew Heron at Imperial College, London, UK [9].

## 3. Results and discussion

**Figure 3.**
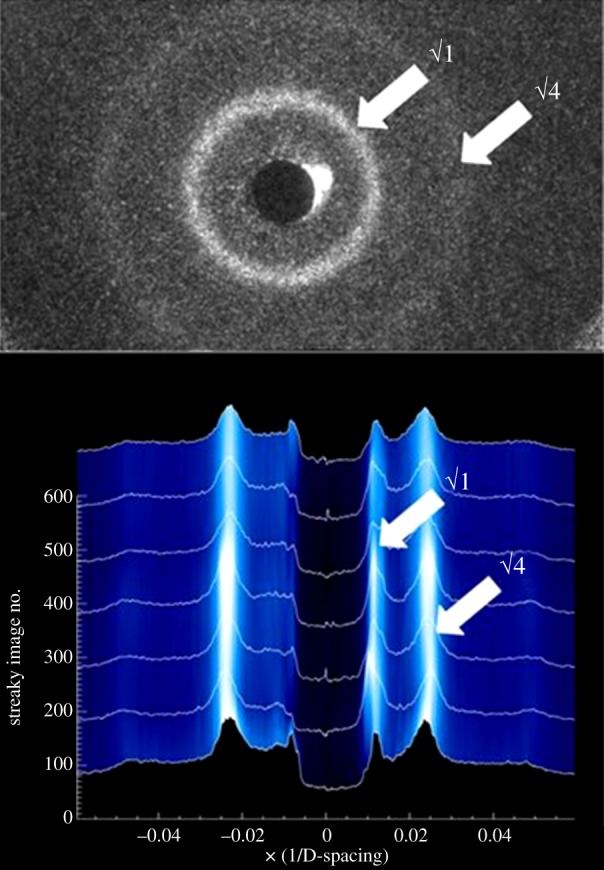
The small-angle X-ray scatter pattern generated by DOPC containing 5 mol% raclopride (top); the integral of the pattern monitored over a period of 500 h. (Online version in colour.)

Prior to probing the effect of raclopride-mediated hydrolysis of membranes of varying lipid compositions, experiments were undertaken to analyse the dose–response curve of raclopride in the standard model membrane system, condensed-phase DOPC. In line with previously reported studies [9,11], experiments were carried out with 5 mol% raclopride as it produced a statistically significant, measurable rate of hydrolysis and no morphological disruption of the host membranes as measured by SAXS, as shown in figure 3.

**Figure 4.**
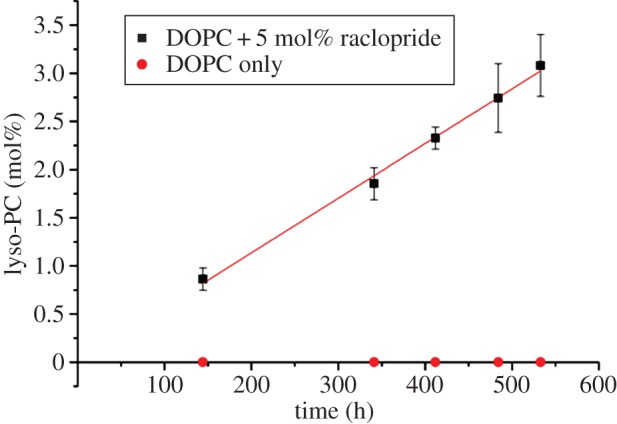
The mean data obtained from nine identical assays studying the hydrolysis of DOPC by raclopride tartrate, prepared and run simultaneously. The trend-line has equation *y =* 0.0057*x*, which is equivalent to a rate of 0.1136 mol h^−1^ mol_RAC_^−1^; error bars are equal to ±2 s.d. of experimental data. Control values show identical DOPC suspension in the absence of raclopride tartrate. (Online version in colour.)

To probe the reliability of the condensed-phase results, nine independently prepared vials containing DOPC and raclopride were assayed and their results compared (figure 4). Data analysis generated a hydrolysis rate of 0.1308 mol h^−1^ mol_RAC_^−1^ (*p* < 1×10^−4^, *r*^2^ = 0.985), and error bars derived from the standard deviation of the data which were used to validate all subsequent assays. Lipid hydrolysis was only observed upon addition of raclopride, confirming that the drug-catalysed reaction was significantly faster than any background degradation processes.

### 3.1. Effect of acyl chain saturation on lipid hydrolysis by raclopride

In order to investigate the effects of saturated lipids within the fluid membrane with respect to raclopride-mediated lipid hydrolysis, DOPC bilayers were doped with lipids of increasing saturated chain lengths. DOPC was chosen as the host lipid to allow direct comparison of results with previous studies [9,11], whereas dopant lipids were selected to provide a congruent set of naturally occurring species, isolating the contributions of the acyl region to the rates of reaction.

These included a range of fully saturated PC lipids: 1,2-dilauroyl-*sn*-glycero-3-phosphocholine (DLPC), 1,2-dimyristoyl-*sn*-glycero-3-phosphocholine (DMPC) and 1,2-palmitoyl-*sn*-glycerol-3-phosphocholine (DPPC). These lipids contain fully saturated chains of 12, 14 and 16 carbons, respectively, and exhibit an increasing gel–fluid transition temperature. DLPC becomes fully liquid at *ca* 8°C, and DMPC at 22–24°C, whereas pure DPPC melts slightly above 40°C [14]. DLPC, DMPC and DPPC were all mixed with DOPC and prepared as previously described with 5 mol% raclopride tartrate.

Experiments were also conducted using mixtures of 1-palmitoyl-2-oleoyl-*sn*-glycero-3-phosphocholine (POPC) with DOPC. This lipid carries one saturated C_16_ chain and one mono-unsaturated C_18_ chain, and unlike some of the saturated species [15,16] mixes ideally with DOPC at all ratios, forming a stable fluid lamellar structure above its chain-melting point of −5°C [17]. Under these experimental circumstances, it was capable of isolating the contribution of the fatty acid tail group in determining the rate of the reaction with respect to choice of acyl group and membrane fluidity. Finally, in order to probe the influence of chain packing efficiency (as typified by the melting temperature) upon the rates of reaction, the hydrolysis of 1-stearoyl-2-oleoyl-*sn*-glycero-3-phosphatidylcholine (SOPC) and the wholly synthetic 1,2-tridecanoyl-*sn*-glycero-3-phosphatidylcholine (C_13_ PC) were also investigated.

**Figure 5.**
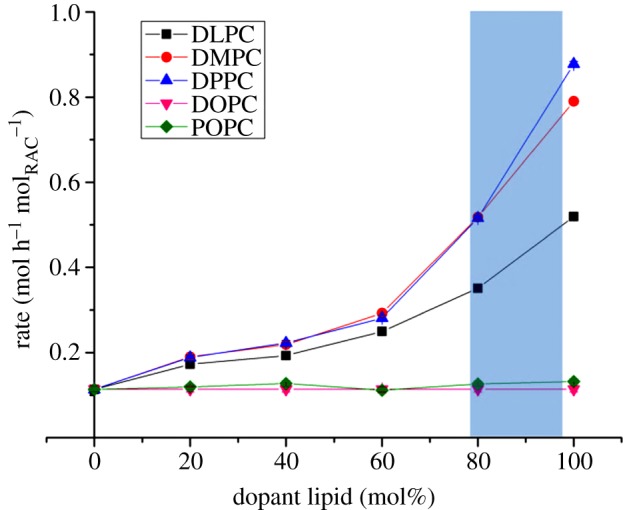
The relationship between saturated-chain lipid concentrations in the membrane and the rate of its hydrolysis by raclopride tartrate. Remaining proportion of membrane was composed of DOPC. Error bars are derived from figure 4 and are smaller than data markers. Shaded region highlights the region of domain formation in DOPC–DPPC systems, as identified by fluorescence methods [15] and X-ray diffraction [16]. (Online version in colour.)

In each of the experiments, lyso-PC was formed at a linear rate dependent upon the system composition, in a similar manner to the data in figure 4. The same trends were visible across the three lipid types (figure 5): hydrolysis in mixtures containing 20, 40 and 60 mol% saturated lipid was very close to that observed in pure DOPC. Above this threshold, hydrolysis began to occur very rapidly and these rates of hydrolysis appear to increase with increasing acyl chain length, up to an eightfold increase in the case of DPPC.

The DOPC–POPC system was tested following the same protocol as described above. Owing to similarities in chemical structure between the two potential lysolipids coupled with their propensity to isomerize into the *sn*-1 form [18], the DOPC and POPC reaction products co-eluted, thus the reaction's chemoselectivity could not be ascertained. However, these data strongly indicate that raclopride-mediated hydrolysis is within error the same for DOPC and POPC lipids, as the observed rates of hydrolysis were independent of the level of POPC in the bilayers (figure 5).

Absolute rates (0.1136 ± 0.00645 mol h^−1^ mol_RAC_^−1^) measured for DOPC/POPC lipid mixtures were identical under the specified experimental conditions; SAXS data (not shown) confirmed that no bulk mesophase changes were detected during the experimental period.

While the hydrolysis data presented in figure 5 also indicate a trend of increase in rate with acyl chain length, there is an obvious and significant discontinuity between the rates of formation of lysolipid species in mixtures dominated by unsaturated lipids relative to those comprising mostly saturated species. Temperature- and composition-dependent phase coexistence regions have been observed between ordered domains enriched in saturated lipids, and fluid-disordered regions enriched in unsaturated molecules. In the case of DOPC–DPPC mixtures at 37°C, the coexistence region has been found by fluorescence methods [15] and X-ray diffraction [16] to form just below 80 mol% DPPC, which agrees well with the experimental data presented above. Although this effect has not been observed in the more fluid DLPC and DMPC systems, the imperfect mixing between the lipid species and the wide disparity between their chain-melting temperatures suggests that something more dynamic and short-lived, but similar, may occur in these shorter chain species.

**Figure 6.**
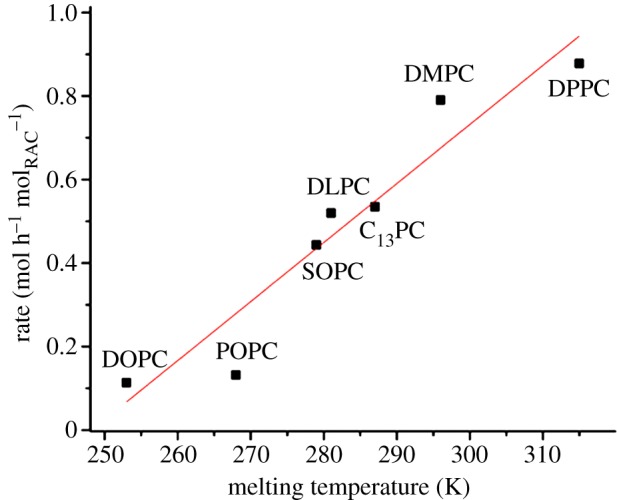
The correlation between a phosphatidylcholine lipid's rate of hydrolysis by 5 mol% raclopride tartrate and its chain-melting temperature, after incubation at 37°C with 3 : 1 PBS w/w. The line of fit is equal to *y =* 0.0141*x −* 3.510, added as a guide for the eye; errors are calculated from figure 4 and are smaller than data markers. (Online version in colour.)

A striking observation that can be made from the data above is the strong, positive correlation between the melting transition temperature of the lipids and the rate at which they are consumed by raclopride-mediated hydrolysis. Furthermore, this trend shows no discontinuity across the *L_*β*_ → L_*α*_* phase transition. To strengthen the confidence in this association, hydrolysis experiments were undertaken using 5 mol% raclopride in two further lipids with chain-melting transition temperatures intermediate between those already established. These were SOPC and C_13_ PC, described previously in figure 2, with melting temperatures of 6°C and 14°C, respectively. The data generated in these experiments fitted the trend closely, as shown in figure 6.

### 3.2. Potential mechanisms

These experimental findings indicate that the hydrolysis of phosphatidylcholine lipids by raclopride is proportional to the chain-melting temperature of the lipid aggregate. The explanations for the observed effects cannot at this stage be conclusively identified: the chain-melting temperature is a complex phenomenon, related to the physical and electronic shapes of the component lipids, the viscosity of the membrane, its surface topology and a number of other factors. A number of hypotheses present themselves, some of which are outlined below; experiments are ongoing to determine which of these predominate in both laboratory and biological conditions.

The introduction of the saturated lipid species, with their relatively high transition temperatures, causes phase separation and eventual bulk phase changes to the system and have a number of implications. It has been demonstrated that amphiphilic compounds experience a log *P* lower by an order of magnitude or more when lipids are in the gel phase compared with the fluid lamellar (although this depends to some degree on the structure of the dopant) [19]. This suggests that while log *P* must contribute to the overall rate, as some degree of membrane penetration is prerequisite for the reaction to occur, it is not the sole rate-determining factor in the reaction as gel-phase systems reacted significantly faster than those in *L_*α*_*.

There is also evidence that shorter-chained lipids are better at sequestering small amphiphilic molecules than longer lipids when both are in the fluid phase [20], presumably because of the greater free volume between chains creating more space for the incorporation of dopant molecules. It has been demonstrated that the addition of fatty acids to such membranes will decrease the transition temperature under all pH conditions below their *p*K_a_ in the membrane (around 7.7) [21] by intercalating between lipid molecules (thus affecting interhead-group interactions) [22] and introducing anionic charge to the interface. This, in turn, will change the partition function and membrane topography experienced by any amphiphile such as raclopride.

However, the regiochemistry of the system is also important. If the rate-determining step of the hydrolysis reaction is the protonation of the lipids’ ester groups, then the two species must necessarily be in close proximity for the process to occur. It is hypothesized that a dominant factor determining the rate of lipid hydrolysis in these systems is linked to the depth within the membrane at which the CAD resides, and that this is determined largely by the size and population of free volume voids within the hydrophobic core of the membrane.

The strength of the interchain forces controls the formation or otherwise of voids, packing defects within the hydrophobic core of the membrane [23], and it is suggested that these control the depth to which raclopride partitions into the membrane. For the reaction to progress, the free proton carried by the CAD must be in close proximity to the ester moieties of the lipid membrane: here, we propose that these voids promote the migration of the CAD towards the hydrophobic centre of the membrane, into a region where they are effectively sequestered and passivated.

The lack of change in the SAXS images to the administration of raclopride was confirmed via fluorescence experiments with the surface charge density probe *N*-(fluorescein-5-carbonyl)-1,2-dioleoyl-*sn*-glycero-3-phosphoethanolamine FPE [24] (not shown), which showed that despite the hydrolytic effects of the drug at a molecular level it had limited structural effect upon the membrane in the head-group region, demonstrating that the electronic and steric influence of the drug was minimal. This suggests that raclopride forms either an non-ionized, hydrophobic species or some form of net-neutral zwitterion or internal salt species [25], making its incorporation into the membrane interior much more favourable if the space is available to accommodate it.

In this model, raclopride acts more like a phase transfer agent, forming only transiently charged species which reside predominantly below the water interface. The more disordered and fluid the membrane, the greater the free volume available to sequester the drug molecules in the membrane core, where they are unable to either acquire protons or deliver them to the lipids’ acyl regions. It has not been possible to definitively identify this mechanism as that underpinning the observed phenomena and indeed it may be the case that they result from a complex combination of these and other effects: however, the combination of drug sequestration into lipid void volumes and a transiently cationic drug present a plausible hypothesis around which further studies will be based.

## 4. Conclusion

The above study demonstrates that small changes in composition between biological membranes may have dramatic effects upon the behaviour of an amphiphile with its constituent lipids. If this behaviour plays a significant role in determining the systemic transport of such species, as postulated by Baciu *et al*. [9], such differences could determine a compound's success and failure in clinical trials. The identification of a consistent, reproducible and linear relationship between the composition and rate of digestion of a membrane also provides, for the first time, a testable explanation for the structural effects some drug molecules have been observed to exert upon their surrounding tissue upon prolonged administration. A number of CAD species cause lipid storage disorders such as phospholipidosis within cells [26]: it may be that this and related conditions are as much a function of the drugs’ direct interactions with the membranes’ lipid matrix as it is with any family of proteins. These interactions might be directly probed *in vitro* using cell cultures incubated in media of differing lipid content; any differences in membrane morphology between cultures can then be attributed to the differences between those membrane compositions rather than any other factor, although it may not be possible to generate quantitative information in the manner of this study from such experiments.

The complexity of the trends described in this study highlights some of the difficulties in modelling or attempting to predict the behaviour of the diverse and highly dynamic environment of biological membranes. By studying these reactions in condensed phase systems where reaction rates are considerably slower than fluorescence-based studies in giant vesicles [9], it is possible to quantify the rates with respect to systematic changes in bilayer composition on sufficiently large populations to produce statistically relevant data. These experiments provide an effective method to probe and quantify drug–lipid interactions, thus characterizing from a chemical perspective a reaction which can be applied to more complex biological systems. They also highlight the importance of probing drug–lipid interactions and the role these interactions have in drug administration and systemic transport.

## Data accessibility

All lysolipid concentration measurements are available via Figshare [27].

## Funding statement

This work was supported by EPSRC grant nos. EP/I017887/1 and EP/G00465X/1 and Life Science Interface Centre for Doctoral Training grant no. EP/E50163X/1; D.C. was part-funded by CASE award S3179 by GlaxoSmithKline PLC.

## References

[1] Seddon AM Casey D Law RV Gee A Templer RH Ces O 2009 Drug interactions with lipid membranes. Chem. Soc. Rev. 38, 2509–2519. (10.1039/b813853m) 19690732

[2] Moussatova A Kandt C O'Mara ML Tieleman DP 2008 ATP-binding cassette transporters in *Escherichia coli*. Biochim. Biophys. Acta 1778, 1757–1771. (10.1016/j.bbamem.2008.06.009) 18634750

[3] Poole RJ 1978 Energy coupling for membrane transport. Annu. Rev. Plant Physiol. 29, 437–460. (10.1146/annurev.pp.29.060178.002253)

[4] Paulsen IT 2003 Multidrug efflux pumps and resistance: regulation and evolution. Curr. Opin. Microbiol. 6, 446–451. (10.1016/j.mib.2003.08.005) 14572535

[5] Lipinski CA Lombardo F Dominy BW Feeney PJ 1997 Experimental and computational approaches to estimate solubility and permeability in drug discovery and development settings. Adv. Drug Deliv. Rev. 23, 3–25. (10.1016/S0169-409X(00)00129-0) 11259830

[6] Overton CE 1899 On the general osmotic properties of the cell, their probable origin, and their significance for physiology. Vierteljahrsschr Naturforsch Ges Zurich 44, 88–135.

[7] Rosso L Gee AD Gould IR 2008 Ab initio computational study of positron emission tomography ligands interacting with lipid molecule for the prediction of nonspecific binding. J. Comput. Chem. 29, 2397–2405. (10.1002/jcc.20972) 18442082

[8] Price DA Blagg J Jones L Greene N Wager T 2009 Physicochemical drug properties associated with *in vivo* toxicological outcomes: a review. Expert Opin. Drug Metab. Toxicol. 5, 921–931. (10.1517/17425250903042318) 19519283

[9] Baciu M 2006 Degradative transport of cationic amphiphilic drugs across phospholipid bilayers. Phil. Trans. R. Soc. A 364, 2597–2614. (10.1098/rsta.2006.1842) 16973478

[10] Sessa G Weissmann G 1968 Phospholipid spherules (liposomes) as a model for biological membranes. J. Lipid Res. 9, 310–318. 5646182

[11] Casey DR Sebai SC Shearman GC Ces O Law RV Templer RH Gee AD 2008 Formulation affects the rate of membrane degradation catalyzed by cationic amphiphilic drugs. Ind. Eng. Chem. Res. 47, 650–655. (10.1021/ie071265q)

[12] Herron AJ 2006 Stored curvature elastic stress in lipid membranes and its effect upon the activity of CTP. London, UK: Imperial College London.

[13] Huang TC Toraya H Blanton TN Wu Y 1993 X-ray powder diffraction analysis of silver behenate, a possible low-angle diffraction standard. J. Appl. Crystallogr. 26, 180–184. (10.1107/S0021889892009762)

[14] Koynova R Caffrey M 1998 Phases and phase transitions of the phosphatidylcholines. Biochim. Biophys. Acta Rev. Biomembrane 1376, 91–145. (10.1016/S0304-4157(98)00006-9) 9666088

[15] Lentz BR Barenholz Y Thompson TE 1976 Fluorescence depolarization studies of phase transitions and fluidity in phospholipid bilayers. 2. Two-component phosphatidylcholine liposomes. Biochemistry 15, 4529–4537. (10.1021/bi00665a030) 974074

[16] Furuya K Mitsui T 1979 Phase transitions in bilayer membranes of dioleoyl-phosphatidylcholine/dipalmitoyl-phosphatidylcholine. J. Phys. Soc. Jpn. 46, 611–616. (10.1143/JPSJ.46.611)

[17] De Kruyff B Demel RA Slotboom AJ Van Deenen LLM Rosenthal AF 1973 The effect of the polar headgroup on the lipid-cholesterol interaction: a monolayer and differential scanning calorimetry study. Biochim. Biophys. Acta Biomembrane 307, 1–19. (10.1016/0005-2736(73)90020-5) 4711186

[18] Wolfenden R Rammler DH Lipmann F 1964 On the site of esterification of amino acids to soluble RNA. Biochemistry 3, 329–338. (10.1021/bi00891a006) 14155094

[19] Høyrup P Davidsen J Jørgensen K 2001 Lipid membrane partitioning of lysolipids and fatty acids: effects of membrane phase structure and detergent chain length. J. Phys. Chem. B 105, 2649–2657. (10.1021/jp003631o)

[20] Luxnat M Galla H-J 1986 Partition of chlorpromazine into lipid bilayer membranes: the effect of membrane structure and composition. Biochim. Biophys. Acta Biomembrane 856, 274–282. (10.1016/0005-2736(86)90037-4) 3955043

[21] Fernández MS González-Martínez MT Calderón E 1986 The effect of pH on the phase transition temperature of dipalmitoylphosphatidylcholine-palmitic acid liposomes. Biochim. Biophys. Acta Biomembrane 863, 156–164. (10.1016/0005-2736(86)90255-5) 3790556

[22] Mabrey S Sturtevant JM 1977 Incorporation of saturated fatty acids into phosphatidylcholine bilayers. Biochim. Biophys. Acta Lipids Lipid Metab. 486, 444–450. (10.1016/0005-2760(77)90094-7) 856286

[23] Wohl CJ Helms MA Chung JO Kuciauskas D 2006 Phospholipid bilayer free volume analysis employing the thermal ring-closing reaction of merocyanine molecular switches. J. Phys. Chem. B 110, 22 796–22 803. (10.1021/jp065406y) 17092030

[24] Cladera J O'Shea P 2001 Generic techniques for fluorescence measurements of protein-ligand interactions; real-time kinetics & spatial imaging. In Protein–ligand interactions. Vol. 2. Structure and spectroscopy (eds Harding SE Chowdhry BZ), pp. 169–200. Oxford, UK: Oxford University Press.

[25] Tsai RS Carrupt P-A Testa B Gaillard P El Tayar N Hoegberg T 1993 Effects of solvation on the ionization and conformation of raclopride and other antidopaminergic 6-methoxysalicylamides: insight into the pharmacophore. J. Med. Chem. 36, 196–204. (10.1021/jm00054a002) 8423592

[26] Reasor MJ Hastings KL Ulrich RG 2006 Drug-induced phospholipidosis: issues and future directions. Expert Opin. Drug Safety 5, 567–583. (10.1517/14740338.5.4.567) 16774494

[27] Casey D Charalambous K Gee A Law RV Ces O 2013 Lipid hydrolysis by raclopride tartrate. See http://figshare.com/articles/Lipid_hydrolysis_by_raclopride_tartrate/852971.

